# Antibiotic resistome from the One-Health perspective: understanding and controlling antimicrobial resistance transmission

**DOI:** 10.1038/s12276-021-00569-z

**Published:** 2021-03-01

**Authors:** Dae-Wi Kim, Chang-Jun Cha

**Affiliations:** 1 Division of Life Sciences, Jeonbuk National University, Jeonju, 54896 Republic of Korea; 2 Department of Systems Biotechnology and Center for Antibiotic Resistome, Chung-Ang University, Anseong, 17546 Republic of Korea

**Keywords:** Bacterial infection, Bacterial genes

## Abstract

The concept of the antibiotic resistome was introduced just over a decade ago, and since then, active resistome studies have been conducted. In the present study, we describe the previously established concept of the resistome, which encompasses all types of antibiotic resistance genes (ARGs), and the important findings from each One-Health sector considering this concept, thereby emphasizing the significance of the One-Health approach in understanding ARG transmission. Cutting-edge research methodologies are essential for deciphering the complex resistome structure in the microbiomes of humans, animals, and the environment. Based on the recent achievements of resistome studies in multiple One-Health sectors, future directions for resistome research have been suggested to improve the understanding and control of ARG transmission: (1) ranking the critical ARGs and their hosts; (2) understanding ARG transmission at the interfaces of One-Health sectors; (3) identifying selective pressures affecting the emergence, transmission, and evolution of ARGs; and (4) elucidating the mechanisms that allow an organism to overcome taxonomic barriers in ARG transmission.

## Antimicrobial resistance: Mapping the spread among humans, animals and the environment

A deeper understanding of the interplay between the human microbiome and micro-organisms present in animals and the environment will be critical for controlling antimicrobial resistance. Researchers have long known that naturally occurring antibiotic resistance genes (ARGs) can transfer between microbial species, disrupting clinical or veterinary antibiotic use. A review by Chang-Jun Cha at Chung-Ang University, Anseong, and Dae-Wi Kim, Jeonbuk National University, Jeonju, South Korea, highlights the importance of a holistic approach known as the ‘One Health’ model to ARG research. The authors explore known sources of ARGs, including soil- and water-dwelling microorganisms, and the pathogens and commensal species found in livestock, wild animals, and humans.The detailed mechanisms of ARG transmission are not fully understood, and the authors stress the need for cutting-edge genome sequencing techniques and other tools to increase understanding.

## Introduction

Since the discovery of the first antibiotic penicillin^1^, antibiotics have been considered essential drugs for treating bacterial infections; however, unlike with other medicines, antibiotic drug resistance has become a great concern owing to its continuous emergence and rapid dissemination among pathogens, as reported in recent cases, posing substantial clinical threats^2,3^. Since the use of early-date antibiotics, such as penicillin and salvarsan, emergence and dissemination of resistance have been observed after introducing antibiotics into clinical settings^4,5^; this resistance is considered to be mainly mediated by antibiotic resistance genes (ARGs). In 1973, environmental ARGs were characterized to be genetically similar to clinical ARGs, suggesting that these clinical genes originated from antibiotic-producing actinomycetes^6^. Consequently, after this discovery, ARGs were intensively studied in only clinical pathogens for >30 years until the resistome concept was proposed^7^; this concept has provided exceptional insights into the origin and dissemination of ARGs. This review aims to describe the concept of the antibiotic resistome; summarize the information from the last decade regarding approaches under the concept; and provide a comprehensive understanding of the origin, emergence, dissemination, and evolution of ARGs. In particular, resistome studies from the One-Health (Human–Animal–Environment) perspective will be essential for deciphering the complex resistome structure and determining prioritized factors to aid in the mitigation of ARG transmission to the clinic.

## Concept of the antibiotic resistome

**Fig. 1:**
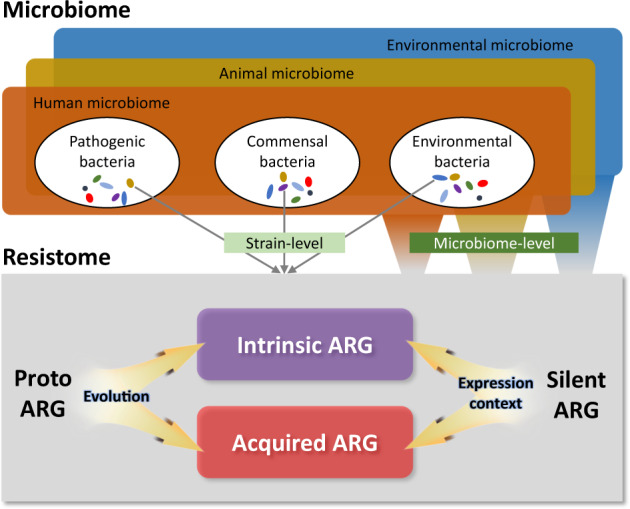
Concept of the antibiotic resistome from the One-Health perspective.

The term “antibiotic resistome” was first coined in 2006 by Gerry Wright’s group, who defined the soil resistome as “resistance determinants present in the soil”, thereby demonstrating that multidrug resistance in a population of environmental bacteria was more prevalent than previously assumed^7^. Later, the resistome was defined as “a collection of all the ARGs and their precursors in pathogenic and nonpathogenic bacteria”^8^. Its constituents were precisely described as “all ARGs, including those circulating in pathogenic bacteria, antibiotic producers, and benign nonpathogenic bacteria”^9^. Considering the origin of ARGs, the resistome has been suggested to include protoresistance genes as a deep reservoir of ARG precursors, as well as clinical, environmental, and intrinsic resistance genes^10^. After a large number of studies on the resistome were published, the definition was further refined by designating types of resistance, such as acquired resistance (vertically or horizontally transferred, taxa-nonspecific), intrinsic resistance (only vertically transmitted, taxa-specific), silent/cryptic resistance (phenotypically sensitive, functional but not expressed), and protoresistance (phenotypically sensitive, little/no activity until mutated)^11^. In summary, the antibiotic resistome encompasses all types of ARGs (acquired and intrinsic resistance genes), their precursors, and some potential resistance mechanisms within microbial communities that require evolution or alterations in the expression context to confer resistance (Fig. 1).

Since 2006, critical information about antimicrobial resistance (AMR) has been revealed based on the resistome concept. Old speculations were confirmed, and critical new findings were suggested as follows: (i) AMR is ancient and ubiquitous in various microbiomes^12–14^; (ii) the antibiotic resistome is complex and diverse^15,16^; (iii) the environmental resistome is the origin and a reservoir of ARGs^13,15,17^; (iv) the resistome is generally determined by the microbial community structure in the natural environment^18^; (v) anthropogenic activities shape the environmental resistome^19^; (vi) mobile genetic elements are responsible for ARG transmission^20^; and (vii) ARGs flow among humans, animals, and the environment^21^. These discoveries have provided the base for recent approaches aimed at improving the understanding of AMR in the human, animal, and environmental sectors, namely, the “One-Health” approach.

## Antibiotic resistome in One-Health sectors

### The One-Health concept

As mentioned above, ARGs circulate among the microbiomes of humans, animals, and the environment, which form the different sectors of the One-Health concept. One-Health is defined as the “collaborative effort of multiple disciplines–working locally, nationally, and globally–to attain optimal health for people, animals, and the environment through policy, research, education, and practice”^22,23^. Initially, zoonoses were recognized as major issues in both humans and animals. In 2008, the importance of “ecosystems” was particularly emphasized in the One-Health concept; this was documented by consultation from several organizations including the Food and Agriculture Organization (FAO), World Organization for Animal Health (OIE), and World Health Organization (WHO) (http://www.fao.org/3/aj137e/aj137e00.htm). In general, infectious diseases, including zoonoses, are the main concerns and targets of the One-Health approach. AMR, which is directly related to such diseases, was considered one of the three One-Health priorities during the tripartite (FAO-OIE-WHO) meeting in 2011^24^. Considering ARG flow among the One-Health sectors, it has been recognized as one of the main issues in the One-Health approach^25,26^. The One-Health concept has focused on interconnections and interdependencies among sectors at local sites; however, recently, considering global health, the comprehension of communication among local ecosystems and the identification of factors that stimulate the global AMR crisis has also gained immense attention^27^. In the present study, we review the achievements in each sector and evaluate the factors that need to be prioritized to mitigate ARG transmission to the clinic.

### Environmental resistome

Since the resistome concept originated in the soil environment, the environmental resistome has been extensively deciphered^7^. ARGs, as ancient and ubiquitous components of bacteria, exist in all ecological niches that harbor various microbial communities^13,28^. From the viewpoint of microbial ecology, the environment can be classified into natural and built environments, and the former can be divided into aquatic and terrestrial environments. The aquatic environment includes marine ecosystems (oceans and estuaries) and freshwater ecosystems (rivers, lakes, and wetlands). The terrestrial environment includes all kinds of terrestrial ecosystems in various climate zones such as forests, deserts, grasslands, and tundra. Built environments include wastewater treatment plants (WWTPs), agricultural sites, aquaculture operations, and hospital environments. In this review, we focus on the environmental resistomes in soil, rivers, WWTPs, and agriculture and aquaculture operation sites.

#### Soil resistome

Numerous studies have demonstrated that the natural soil environment is the origin of ARGs^13,17,29^. Many novel ARGs have been characterized from the soil environmental isolates or metagenomic sequences^30–32^. The presence of complex resistomes in permafrost and an isolated cave indicates that the environment is indeed the origin of ARGs^12,14^. The natural soil environment is regarded as a major reservoir of the antibiotic resistome, including intrinsic resistance and acquired resistances genes^33^; however, only some of these ARGs can acquire mobility and subsequently emerge in clinical settings, considering that mobile genetic elements (MGEs) syntenic with ARGs are rare in soil compared with pathogens^18^.

#### River resistome

Freshwater environments, including rivers, are considered reservoirs and dissemination routes for AMR^34^. Several studies have clearly depicted a larger increase in ARGs in human-impacted river sites than in the pristine river sites, indicating the effects of anthropogenic activities on the river resistome^35–37^. The river resistome is important for the potential reintroduction of AMR in humans because rivers are a major source of drinking water and irrigation water for agriculture^38,39^. Furthermore, in riverine ecosystems, the presence of novel ARG contexts, implying the evolution and adaptation of ARGs for transmission, can cause significant challenges when they are reintroduced into human populations^40,41^. The plausible factors influencing the river resistome have been analyzed in the contexts of the effects of pollution sources and the statistical evaluation of critical factors among various anthropogenic activities. Regarding pollution sources, the effects of WWTPs on the river resistome have been surveyed in various river systems because WWTPs are considered major pollution sources in rivers in urban regions^42^. In general, assessments of resistomes in upstream and downstream regions of particular river sites, to which WWTP effluents are exposed, revealed that effluents certainly affect the diversity and abundance of the river resistome^43–45^. For statistical elucidation, correlations between the river resistome and various factors were evaluated. Antibiotic residues, microbial community structures, environmental parameters, and socioeconomic factors related to anthropogenic activities were found to be associated with ARG increases and alterations in the diversity of the river resistome^37,46,47^. Moreover, fecal pollution has been identified as one of the major contributors to changes in the river resistome by tracking the genetic markers of human fecal contamination, such as crAssphage or fecal specific bacterial taxa^37,48,49^. However, a recent study reported that fecal contamination could not fully explain the dramatic ARG increase downstream of a heavily urbanized river, thereby indicating the proliferation of ARGs in the river environment via MGEs^37^. Unlike the natural soil resistome, the river resistome subjected to anthropogenic activity is not determined by the whole microbial community, indicating the prevalence of horizontal gene transfer (HGT) of ARGs among certain bacterial hosts^18,37,50^. Recent studies have also revealed a correlation between genetic traits and resistome changes, such as MGEs for HGT and metal resistance genes (MRGs) co-selected with ARGs, and the environmental hosts in which ARGs proliferate^37,43,44,49,51,52^. Among the MGEs, class 1 integrons have been demonstrated to be important molecular markers for resistome proliferation and anthropogenic pollution^53,54^. Some ARGs have been suggested as core resistome or critical ARGs responsible for altering the river resistome^37^. To prioritize critical factors affecting the river resistome, generalized approaches at the global scale and consolidation of the present river resistome data are required^55^.

#### Wastewater and WWTP

Wastewater and WWTPs are considered hotspots for the proliferation of antibiotic-resistant bacteria (ARB) and ARGs^56^. They are among the major interfaces between humans and the environment^56^ and are also recognized as hotspots for HGT of ARGs, resulting in the dissemination of AMR^57^. The presence of antibiotics, metals, and disinfectants in wastewater and WWTPs could act as selection pressures for ARG transfer and co-selection of ARGs with MRGs and disinfectant resistance genes, even though their concentrations are remarkably lower than therapeutic concentrations^58^. Recently, the mobile resistome in WWTPs comprised a major proportion of the WWTP resistome, resulting in the identification of critical ARGs in such environments^59^. The role of wastewater as a reservoir of ARGs is gaining substantial attention, considering the continuous reports on novel ARGs (some of which are already mobile)^60^; moreover, antibiotic-resistant pathogens and ARGs have recently emerged in the clinical setting^61^. The resistome in WWTPs according to the treatment steps has been intensively studied, and the persistence of ARGs throughout the treatment stages was elucidated^62–64^. A long-term investigation of WWTPs revealed remarkable changes in the resistome every 2–3 years, indicating successive selection of the resistome in activated sludge^61^. These efforts have focused on risk assessments of WWTPs; however, the consolidation of WWTP data from various regions and the establishment of standardized methodologies are required to derive the environmental framework for wastewater and WWTP resistomes^56,57^.

#### Agriculture

Previous studies have reported the importance of soil biota under the influence of anthropogenic activities in planetary health systems^33^. Organic manure and reclaimed wastewater have been identified as critical human and animal waste products that can induce alterations in the diversity and prevalence of ARGs and ARB in soil environments^33,65^. Agricultural sites (plant production environments) in which manure and wastewater are frequently used for soil amendment have been recognized as important routes for ARG transmission from humans and animals to the environment^66^. Moreover, introduced ARGs could be reintroduced into humans via agricultural products^67^. The FAO, WHO, and OIE emphasize the significance of the agricultural environment in ARG transmission (“Joint FAO/WHO expert meeting in collaboration with OIE on foodborne AMR: role of the environment, crops, and biocides”, 2019). Furthermore, recent studies have emphasized overall surveillance of ARGs in the agricultural environment and agricultural products to understand and control ARG transmission to humans^68^.

#### Aquaculture

Abundant use of antibiotics as prophylactic and therapeutic agents in aquaculture can induce selection, evolution, and HGT of ARB and ARGs in the environment^69^. Diverse microbiota, highly populated aquacultured animals, and the overuse of antibiotics designate aquaculture a “genetic hotspot” for gene exchange^70^. Several studies have reported an increase in ARGs in aquaculture systems^71,72^. The use of a specific antibiotic could promote ARGs against other classes of antibiotics, thereby indicating co-selection of ARGs mediated by MGEs^72^. Furthermore, the selected ARB and enriched ARGs in aquaculture could affect the microbiota and resistomes of adjacent environments^73^. The incidence of AMR in aquaculture can cause negative economic and social repercussions in these industries^69^. Enriched ARGs could be reintroduced into humans via adjacent environments and aquacultured animal products, suggesting an urgent need for surveillance of AMR.

### Animal resistome

According to the WHO^74^, antibiotics are used in livestock animals for three purposes: therapeutic veterinary medicine, disease prevention (prophylaxis and metaphylaxis), and growth promotion. The latter two are strongly associated with the overuse of antibiotics in animals maintained under crowded conditions. For growth promotion, sublethal concentrations of antibiotics are widely used, although the mechanisms associated with weight gain remain unclear. Recent studies have reported that even low antibiotic concentrations affect resistome expansion in animals^75^. The use of antibiotics in livestock animals could trigger the selection of ARB and ARGs, which could subsequently be transferred to humans^21^.

The resistomes of various livestock animals have been investigated, and some regional studies have revealed a positive correlation between antibiotic use and resistome profiles as well as MGE dependency^76^. A recent study on the resistomes of slaughtered pigs and broilers across nine European countries revealed that the abundance and diversity of the fecal resistomes depended on antibiotic use across the nation^77,78^. In a subsequent and cross-sectional (human–animal) study, surveys of the resistomes of farm and slaughterhouse workers exposed to animals suggested that the human resistome was influenced by the animal resistome, thus, emphasizing the importance of surveillance for AMR in livestock animals^79,80^. Considering the complexity of the animal resistome and the presence of various external factors at livestock sites, systematic approaches should be implemented for further analysis.

In addition to livestock animals, wild animals are also known to be a source of ARG dissemination. The resistome of human-contacted wild animals revealed more-diverse ARGs than that of noncontacted animals^81,82^, thereby indicating that wild animals contribute to ARG transmission between humans and animals. Among wild animals, migratory birds were found to be responsible for ARG dissemination to the environment^83^. Moreover, the presence of pathogens carrying emerging ARGs in companion animals, such as dogs and cats, suggests that these animals act as ARB and ARG reservoirs^84^; however, resistome-level studies on these animals are scarce. Considering the proximity of companion animals to humans in daily life, the resistomes of these animals should be surveyed and assessed from the perspectives of the resistome and the One-Health to control ARG transmission to humans.

### Human resistome

Metagenomics based on next-generation sequencing technology has been applied to the human microbiomes of the gut, skin, and respiratory tract to assess their resistomes. Understanding the dynamics of the human resistome and its relatedness to the other One-Health sectors is essential to control ARG flow from the other sectors to the human sector, particularly ARG transmission to disease-causing bacteria^85^. The resistome of the commensal bacterial community in the human microbiome is regarded as an important reservoir and dissemination route for clinical ARGs^86^. Several studies have elucidated the role of the human gut resistome by deciphering it and comparing its similarity with those of pathogens^17,87,88^. Although the transmission of ARGs from commensal bacteria to pathogens seems to be scarce^89^, the presence of almost identical ARGs and similar genetic contexts between the human gut and pathogenic bacteria indicate the importance of the human gut microbiome in the emergence of clinical ARGs. Correlations between the human gut resistome and the animal gut and environmental resistomes have been characterized^79,90,91^. Worldwide cohort studies revealed a nation-level resistome structure^92,93^, and antibiotic administration played a pivotal role in resistome and mobilome (a collection of all types of MGEs) structures^94,95^. Among the complex human gut resistomes, the importance of the mobile resistome, in which ARGs are associated with MGE in a genetic context, has been emphasized to understand ARG transmission from animals and the environment to pathogens^88,96–98^. Continuous identification of novel ARGs in the human gut proved the role of the human gut as an ARG reservoir and transmission route^99^. Furthermore, the establishment of the human gut resistome in infants, vertical transmission of the resistome and mobilome from mother to infant, and the correlations between the resistome and diseases have been studied to characterize the dynamics of the human gut resistome^95,100–102^.

The resistome of the human respiratory tract was assessed to understand the etiological cause and diagnosis of the AMR profile of infection^103^. In particular, resistance in the microbiome of the respiratory tract in patients with cystic fibrosis and chronic respiratory disease is of great concern due to frequent polymicrobial infection in these patients^103^. Moreover, respiratory tract infection in intensive care unit patients is a critical factor determining patient survival; however, current studies on the resistome of the respiratory tract are scarce, and further systematic approaches are required to characterize the resistome and provide guidance for proper antibiotic administration against such infections. The resistome of human skin of uncontacted Amerindian infants in a neonatal intensive care unit and hands exposed to the public metro system were characterized^104–106^, indicating a potential role of the skin microbiome in ARG transmission.

## Future perspectives

**Fig. 2:**
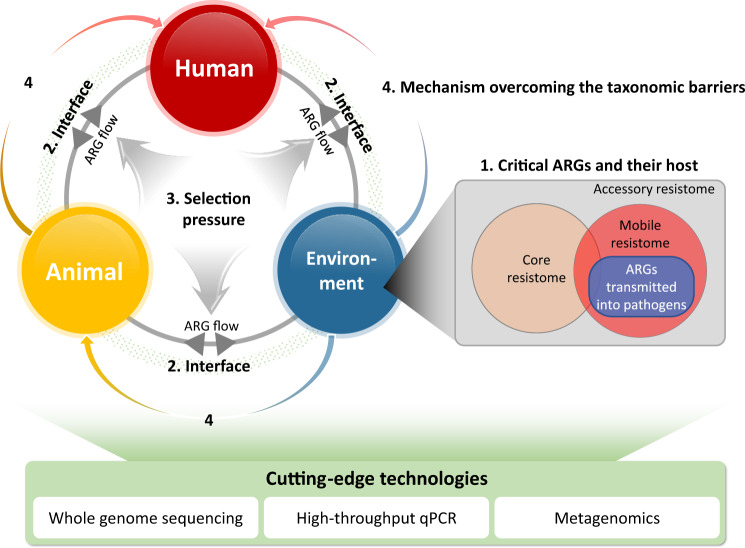
Future perspectives for antibiotic resistome studies with a One-Health approach.

Over the last decade, the paradigm of AMR research has changed, as the origin, transmission, and evolution of AMR have been discovered; however, as ARG transmission to humans from other One-Health sectors is only partially understood, it is expected that in the future, the following research approaches will be conducted based on the foundation of scientific achievements over the last decade. These attempts will improve our understanding of ARG transmission and guide strategies to mitigate AMR dissemination (Fig. 2).

### Applications of cutting-edge technologies for antibiotic resistome studies

The antibiotic resistome has been recognized as dynamic and ever expanding^11^, thereby indicating the importance of cutting-edge technologies to elucidate its dynamics and to explore its diversity in depth. Newly developed next-generation sequencing methods and bioinformatics tools have accelerated the applications of such sequence-based methods for resistome studies in all One-Health sectors^107^. Metagenomics is an essential sequence-based approach to comprehend the complex resistome within the microbiome. Three different approaches, including functional metagenomics, shotgun metagenomics, and targeted gene sequencing (amplicon sequencing), have been applied in several studies, considering the trajectory of the studies over the last decade. Metagenomics has been widely used to identify novel ARGs (functional metagenomics)^99,108^ and ARG variants or genetic contexts of ARGs (targeted gene sequencing)^31,41,109,110^, and to analyze ARGs at the resistome level (shotgun metagenomics)^37,111–113^. According to specific research purposes, appropriate sequencing platforms, ARG databases, and analysis pipelines should be employed to understand AMR at the resistome level. A high-throughput quantitative polymerase chain reaction array, also termed the SmartChip system (Takara), has been widely used for resistome studies in various environmental microbiomes^37,46,114,115^. This system is advantageous, as a high number of ARGs can be analyzed simultaneously within a short time, and this system is more sensitive than the metagenomics approach for detecting ARGs; however, the limitation is that only previously characterized ARGs and MGEs can be detected, and information on the hosts carrying these genetic elements cannot be obtained. In the original primer version^76^, the primers were found to be redundant, and primers for newly discovered ARGs were absent. Recently, Primer set 2.0 was released to solve these issues^116^. Considering the cost, time, and sensitivity, this method can be widely used for resistome-level analysis if the continued development of primer sets is updated. For resistome analysis at the bacterial isolate level, whole-genome sequencing (WGS) for antibiotic susceptibility testing (AST) is gaining importance as a powerful approach to predict antibiotic susceptibility profiles at the genome level; this approach can be aided by machine learning and statistical models, although the inconsistency between the WGS method and culture-based AST needs to be improved^117^. In conclusion, these cutting-edge technologies will be essential for understanding ARG transmission between microbiomes or bacterial strains and will provide important information about the antibiotic resistome in the context of the One-Health approach.

### Ranking the critical ARGs and their hosts

Since a complex resistome exists in all microbiomes, recent studies have attempted to classify particular ARGs that pose great threats to humans and other organisms. Their classification was revealed by understanding the core resistome, which is relatively stable in the microbiome, and the mobile resistome, which is genetically associated with MGEs^37,88,118,119^. Through these approaches, mobile ARGs in the microbiome have been recently reported^119^. Moreover, information about hosts of these mobile ARGs is essential to understand ARG carriers and their roles in transmission to humans^37,96,113^.

### ARG transmission at the interfaces among One-Health sectors

It is important to understand ARG flow between the One-Health sectors by monitoring the interfaces among these sectors^120^. Thus, appropriate configuration of the associated testbeds and their interfaces is essential. If both culture-dependent and culture-independent methods are simultaneously employed in an appropriate manner to understand the resistome at the interfaces, then snapshots of transmission among the One-Health sectors can be obtained. Valuable information integrated into and accumulated through these approaches will reveal the actual dynamics of ARG transmission.

### Selective pressures affecting the emergence, transmission, and evolution of ARGs

Recent studies have reported that anthropogenic activities shape the environmental resistome^37,46,93^; however, limited information about the specific factors that induce changes in the resistomes of One-Health sectors is available. To reveal the selective pressures causing such changes, it is necessary to collect relevant data in a more granular manner, and to compare the data with the resistome data in a reliable statistical way. Furthermore, monitoring changes in the resistome in response to individual selective pressure in microcosm or mesocosm studies are required to obtain experimental evidence. A systematic understanding of the correlation between selective pressure and resistome changes resulting from mitigation will be an essential prerequisite for mitigating ARG transmission to humans.

### Mechanisms overcoming taxonomic barriers in ARG transmission

AMR has long been present in antibiotic-producing bacteria, typically nonpathogenic environmental bacteria^6,7,121^; however, at some point, after crossing over into pathogens or related strains through evolutionary events that crossed taxonomic barriers, ARGs became mobile and emerged in the clinical setting^121^. No clear mechanism has been clearly identified yet, although a case study suggested a “carry-back” mechanism for ARG transfer from antibiotic producers to pathogens^122^. Considering the substantial number of environmental ARGs that are not yet mobile, the mechanisms of taxonomic barrier crossing in ARG transmission need to be better understood.

## Conclusion

The concept of the antibiotic resistome and a One-Health approach is crucial to understand and mitigate ARG transmission between the One-Health sectors. Although important findings of AMR in these sectors have been recently unveiled, various factors need to be explored to understand the origin, emergence, dissemination, and evolution of ARGs. Based on cutting-edge technologies, several research topics suggested in the present study need to be further explored in the near future. Such endeavors will facilitate our hopes to overcome the arms race between antibiotics and AMR.
